# Demand and Congestion in Multiplex Transportation Networks

**DOI:** 10.1371/journal.pone.0161738

**Published:** 2016-09-22

**Authors:** Philip S. Chodrow, Zeyad al-Awwad, Shan Jiang, Marta C. González

**Affiliations:** 1 Operations Research Center, Massachussetts Institute of Technology, Cambridge, MA 02139, United States of America; 2 Center for Complex Engineering Systems, King Abdulaziz City for Science and Technology, Riyadh 11442, Saudi Arabia; 3 Department of Civil and Environmental Engineering, Massachussetts Institute of Technology, Cambridge, MA 02139, United States of America; Universidad de Zaragoza, SPAIN

## Abstract

Urban transportation systems are multimodal, sociotechnical systems; however, while their multimodal aspect has received extensive attention in recent literature on multiplex networks, their sociotechnical aspect has been largely neglected. We present the first study of an urban transportation system using multiplex network analysis and validated Origin-Destination travel demand, with Riyadh’s planned metro as a case study. We develop methods for analyzing the impact of additional transportation layers on existing dynamics, and show that demand structure plays key quantitative and qualitative roles. There exist fundamental geometrical limits to the metro’s impact on traffic dynamics, and the bulk of environmental accrue at metro speeds only slightly faster than those planned. We develop a simple model for informing the use of additional, “feeder” layers to maximize reductions in global congestion. Our techniques are computationally practical, easily extensible to arbitrary transportation layers with complex transfer logic, and implementable in open-source software.

## Introduction

Multiplex network analysis [1–11] has emerged as an important method for extracting system-level insights from multimodal transportation systems [12–15]. Existing studies have treated such systems with an exclusive focus on the topology and geometry of transportation infrastructure, analyzing linkages between road networks, subway maps, bus routes, commuter train lines, or, at a much larger scale, air traffic networks. However, transportation systems are intrinsically sociotechnical, and their behavior is dependent on the interaction between infrastructure and user behavior–especially the structured pattern of user demand.

A standard summary of such demand is the Origin-Destination (OD) matrix, whose entries give the number of travelers between each possible pairing of origin and destination in the city. Until recently, OD matrices were typically prohibitively difficult to construct. As consequence, many existing system-level models of urban transportation make an a priori assumption on the structure of the OD matrix in the absence of data. Typical assumptions are that the OD matrix is uniformly and randomly generated in one [16] or both [17] axes, or that the matrix is uniform and fixed [12, 15]. However, the advent of mobile telephony has enabled passive collection of massive quantities of human mobile traces, which may be used to construct reliable, validated OD matrices [18–20]. The availability of such data has thereby enabled scalable, data-driven, sociotechnical analysis of urban transportation systems [21–26].

Such analysis contributes two layers of realism when compared to uniform and stochastic OD models. First, system-level indicators such as travel time distributions can be more accurately calculated by incorporating basic facts of urban geometry, especially the prefgerence of commuters to live closer to their workplace than would be predicted by uniform random chance [27]. Second, reliable traffic demand data enables the modeling of network congestion, which can dramatically alter travel time estimates for routes that use popular or under-capacitated network links. While congestion is a fundamental fact of modern transportation, it is currently discussed only theoretically in multiplex studies [12, 28].

Our contribution is to use empirical OD demand and the tools of multiplex network analysis in the systems-level study of urban transportation under congested travel. As we show, the incorporation of measured OD demand and congestion effects substantially alters both the quantitative metrics and qualitative behavior of multiplex systems. We focus on two major questions that may arise in planning contexts:

1. How does the introduction of a fully new mode of transportation impact commuting flows at the city level?
2. How can planners prioritize which areas and routes to target for adoption of the metro system, for example by providing “feeder” buses?

Our case study is the city of Riyadh, where the construction of a brand new metro system currently represents one of the largest urban planning projects in the world. We use data on Riyadh’s road network structure, the planned metro network, and OD demand for morning commutes derived from CDR data provided by a major phone service operator. We conduct all analyses in Python, for which we have developed a multiplex module for multiplex transportation analysis with OD matrices.

In order to focus on methodology in addressing these questions, we simplify the multiplex model by choosing not to model waiting times [15] or limited metro capacity. We also make the simplifying assumption that travelers can and do drive both to and from metro stops, rather than, e.g. walking or bicycling. While indeed a simplification, this assumption is relatively realistic in Riyadh, where a lack of pedestrian infrastructure [29] and the rising popularity of ride-hailing services make walking and bicycling unusual. For example, a 1989 study estimates that just 2% of Riyadh citizens walked distances of more than 1km on a daily basis [30], a figure which is likely even smaller today. However, in cities where alternative transportation is more widely used, realistic modeling may require additional multiplex layers for walking, bicycling, or additional public transit.

## Materials and Methods

### From Travel Demand to Network Flows

We constructed Origin-Destination (OD) matrices for the network using Call Detail Record (CDR) data representing 2.2 million in an area comprising a population of 5.8 million. To model the network at its most critical time, the OD matrix used corresponds to morning peak commuting flows between 7:30am and 8:30am. To assign routes based on the OD matrix, we performed a version of Iterative Traffic Assignment. We divided commuters into six groups, the first four containing a uniformly-distributed 20% of total flows, and last two containing 10%. We chose this grouping, rather than the traditional [40%, 30%, 20%, 10%], in order to avoid unrealistic crowding around metro stations for high metro speeds. The first group of 20% was allowed to choose shortest paths through the network based on estimated free-flow travel times. Travel times for each segment were then updated in light of congestion according to the standard BPR formula

$$t_{e}^{*}=t_{e}\left(1+A{\left(\frac{j_{e}}{c_{e}}\right)}^{B}\right),$$ (1)

where *t*_*e*_ is the free flow time on segment *e*, *j*_*e*_ is the flow through *e* on the current iteration, and *c*_*e*_ is the capacity of the segment. *A* and *B* are tunable parameters; following standard practice, we set *A* = 0.15 and *B* = 4. Since the metro is assumed uncapacitated, travel time on those segments is always assumed to be equal to the free flow speed. We then allowed the next group to choose shortest paths through the network based on these updated edge costs, and continued this process until all groups were assigned.

In order to analyze the dependence of multiplex dynamics on the metro layer, we performed the above assignment for varying metro speeds. Following [12], we model the metro as a network with mean speed *v*_*c*_/*β*, where *β* is a variable parameter and *v*_*c*_ = 38km/hr is the mean travel speed on the street layer under congestion in the absence of a metro layer. Lower values of *β* correspond to faster metros. For example, at *β* = 0.5, the metro runs at 76km/hr, which is twice the mean speed of the street layer without a metro. Based on technical specifications for Riyadh and previous measurements of operational metro systems [31], we estimate an average effective metro speed of 47km/h, which corresponds to *β* = 38/47 ≈ 0.8.

### Validation of Assigned Flows

**Fig 1:**
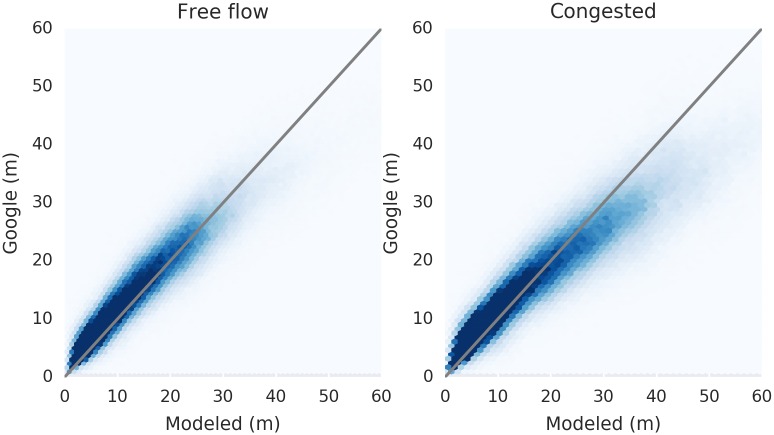
Validation of assigned flows. Our estimated travel times agree quite closely with Google Maps estimates in the free flow case (left). Some curvature is evident in the congested case, suggesting a small amount of systematic disagreement between models.

To ensure the realism of our modeled flows, we compared our travel time estimates to those from Google Maps. Using a web crawler, we queried Google Maps for travel times for the top 679,085 OD pairs by flow volume under free flow and congested scenarios. The comparison between the ITA-estimated travel times and those of Google are shown in Fig 1. We then scaled free-flow street speeds to more closely match without-traffic travel-time estimates based on Google Maps, and used these scaled speeds in subsequent computation. While the resulting match is closer for free flow estimates than congested estimates, we found both to be acceptable for modeling purposes.

## Results

### Assigned Flows

**Fig 2:**
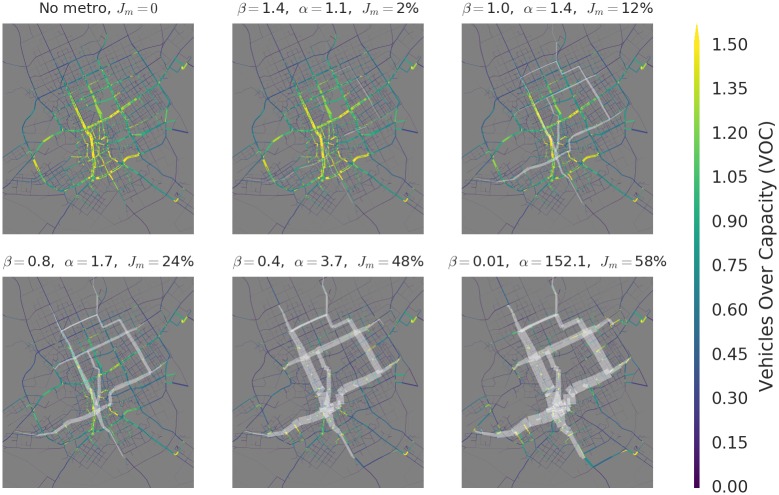
Multiplex flows for varying metro speeds. *J*_*m*_ is the percentage of all person-kilometers which are traveled through the metro network. For slow speeds (higher *β*), congestion is concentrated along major thoroughfares in the downtown area in the center-west. The introduction of the metro at *β* = 1.4 has small impact on flows, handling just 2% of total. As effective speed increases, progressively more flow passes through the metro. Simultaneously, global congestion is reduced, but increases locally near key metro access points under very high speeds. Maps produced using Python’s networkx package [32] v. 1.10, using road network data provided by the Arriyadh Development Authority (ADA).

Fig 2 shows the spatial distribution of flows for varying levels of *β*. At each level of *β*, we have measured the empirical speed ratio *α* between the metro and the streets *after* routes have been assigned through ITA. While *β* is a modeling parameter and can be viewed as the (mean) ratio of “posted speed limits,” *α* is a system measurement and reflects actual speeds experienced due to congestion. The quantity *α* therefore estimates, on average, how attractive the metro might be to commuters given realistic congestion conditions, with higher values indicating greater adoption appeal.

For high *β*, the metro is not substantially faster than the congested street layer, and adoption is very low. Our estimated realistic *β* = 0.8 corresponds to an empirical speed ratio of *α* = 1.7—the metro travels significantly faster than the congested streets, resulting in significant adoption of the metro layer. As *β* decreases, the benefits of using the metro increase, and progressively more travelers use the metro for progressively larger portions of their journeys, reducing congestion in most areas. As previously observed, however, congestion is not just reduced but also dispersed: key roads used to access the metro may become more congested than they were before the metro’s introduction [12].

For very low *β*, we observe the existence of a limiting behavior, in which 58% of total flow occurs on the metro network. Because it persists under further increases in metro speed, the limiting distribution reflects the fundamental geometrical relationship between the metro network and the street network: compared to the street network, the metro network’s spatial reach is limited. The remaining 42% of flow is irreducible by increases in metro speed, and reflects flow due to travel to and from metro stops, or trips for which it would take longer to drive to and from metro stops than it would to drive directly to the destination.

### Quantifying Multiplex Flow Effects

**Fig 3:**
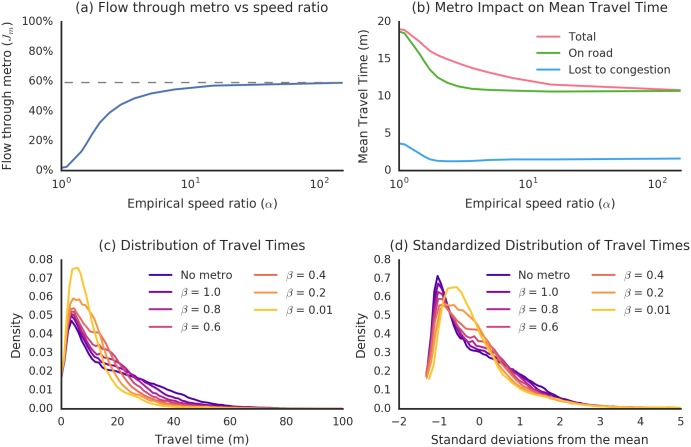
Behavior of multiplex flows with variable metro speed. (a) For very high speed ratios, proportional flow through the metro approaches a limiting value of 58%, reflecting the partial geographic extent of the metro network. (b) As metro speed increases, total travel times decrease monotonically. However, most of the reduction in time spent on the road is achieved for relatively slow metro speeds, indicating that environmental returns to very fast metro schemes may be limited. (c) Dependence of travel time distributions on metro speed. A small number of travelers have very long commutes even for low *β*, corresponding to origins or destinations that are far removed from the metro network. (d) Increasing the metro speed also changes the qualitative structure of travel time distributions, as the metro smooths out heterogeneities introduced by empirical OD travel demand by linking distant areas of the city.

Fig 3 summarizes the behavior of flows under increasing metro speeds. Metro usage increases monotonically with the relative speed *α* of the metro to the street layer; however, this increase approaches an asymptotic boundary of *J*_*m*_ = 58%. The impact on travel times is more complex. Quantitatively, mean travel times reduce monotonically with metro speed down to a minimum of 43% of their original value. However, the amount of time spent driving rapidly levels off: over 85% of the total reduction in time spent on the road is realized when *β* = 0.6, which is 33% faster than our estimated realistic *β* = 0.8. The speed *β* = 0.6 also marks the speed at which maximum reduction in time spent in congestion is achieved. These two factors are of substantial interest for mitigating the environmental impact of urban transit. This result suggests that metro speeds slightly faster than currently planned could produce positive environmental impact, but that there may be diminishing returns to increasing metro speeds beyond that. Determining the precise rate at which returns diminish would require a more detailed transportation model.

Metro speed impacts not only mean travel times, but also their standardized (qualitative) distribution. This qualitative shift reflects the fact that the fast metro fundamentally alters the geometrical structure of the urban transportation network, closely connecting areas that were previously remote.

### Prioritizing Transportation Adoption

The introduction of the metro network tends to reduce global congestion, but also disperse congestion to the periphery for sufficiently high speeds. In practice, cities may be able to further reduce congestion by operating “feeder” networks—such as buses—with the goal of making the main network more accessible to residents throughout the city. Implementing such a network may require substantial resources, however, underscoring the need for efficiency. The multiplex model can also shed light on how areas within the city can be prioritized for such service, with an eye toward reducing total congestion.

#### Quantifying Congestion Contributions

Let **j** be vector whose *e*th component is the flow along edge *e* under a given assignment of flows. Then, the total time lost to congestion under the assignment **j** is

$$T_{c}\left(j\right)=\sum_{e\in E}j_{e}\tau_{e}\left(j_{e}\right)\;,$$ (2)

where $\tau_{e}=t_{e}^{*}-t_{e}$ is the time lost to congestion on a given edge as a function of flow through that edge. We can quantify the impact of small changes in flow on *T*_*c*_(**j**) using the gradient of *T*_*c*_, whose *e*th component is

$${\left[\nabla T_{c}\left(j\right)\right]}_{e}=\tau_{e}\left(j_{e}\right)+j_{e}\frac{\partial \tau_{e}}{\partial j}{|}_{j_{e}}\;.$$ (3)

The vector −∇*T*_*c*_(**j**) may be interpreted as the steepest descent direction in the unconstrained minimization of *T*_*c*_(**j**).

To analyze the impact of changes in flow along a route, let *p* be a path (sequence of edges) through G and let **e**_*p*_ be the vector whose *e*th component is 1 iff *e* ∈ *p*. Then, the quantity

$$\Delta_{p}=-\nabla T_{c}\left(j\right)\cdot e_{p}$$ (4)

approximates the impact of removing a single unit of flow from *p* on the global congestion function *T*_*c*_. Let Δ_*od*_ = Δ_*p*_ if *p* is the shortest path between origin *o* and destination *d*.

#### Congestion-Based Prioritization

**Fig 4:**
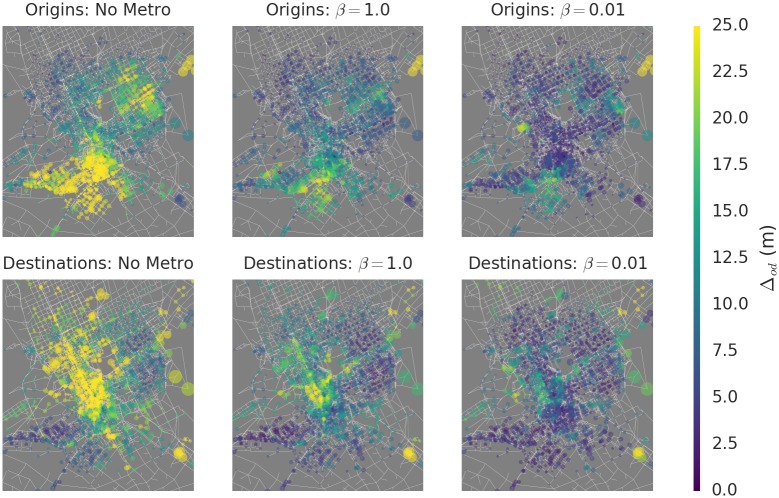
Spatial heterogeneity in contributions to global congestion. The congestion impact Δ_*od*_ is aggregated over *o* (top) and *d* (bottom). The aggregates Δ_*o*_ and Δ_*d*_ can be interpreted as the expected impact of removing one driver who lives (resp. works) at a location from the streets, without knowing the details of their route. Importantly, Δ_*od*_ is highly unevenly distributed throughout the city, indicating opportunities to prioritize those who live in the southwest and northeast, and those who work downtown. The heterogeneity is reduced by faster metro speeds, but does not vanish, and even increases in the limiting case of very low *β*. Maps produced with Python’s networkx package [32] v. 1.10, using road network data provided by the Arriyadh Development Authority (ADA).

Importantly, the distribution Δ_*od*_ is spatially structured and highly heterogeneous. Fig 4 shows the spatial distribution of $\Delta_{o}=\frac{1}{J}\sum_{d}j_{od}\Delta_{od}$—the mean congestion contribution of travelers from origin *o*—and $\Delta_{d}=\frac{1}{J}\sum_{o}j_{od}\Delta_{od}$—the mean congestion contribution of travelers to *d*. It is clear that there are specific regions that make large contributions to global congestion, such as origins in the residential area in the southwest and destinations in the downtown area in the center-west. Furthermore, this spatial structure persists for all levels of *β*, though the magnitude of the differences vary.

The persistent, heterogeneous distribution of Δ_*od*_ presents an opportunity to efficiently reduce global congestion by prioritizing these high-impact origins and destinations for public transportation access. To model such prioritization, we computed the congestion impact Δ_*od*_ for each route, and then deleted the flow in the OD matrix along the 50,000 routes with the highest values of Δ_*od*_. The deleted flow accounts for 15% of the total represented in the data. This deletion models perfect adoption of public transportation by these congestion contributors. We then performed ITA to assign the modified travel demand to the road network. As a comparative baseline, we also deleted 15% of flow from the OD matrix at random, and performed ITA. We repeated this procedure at varying metro speeds as measured by *β*.

**Fig 5:**
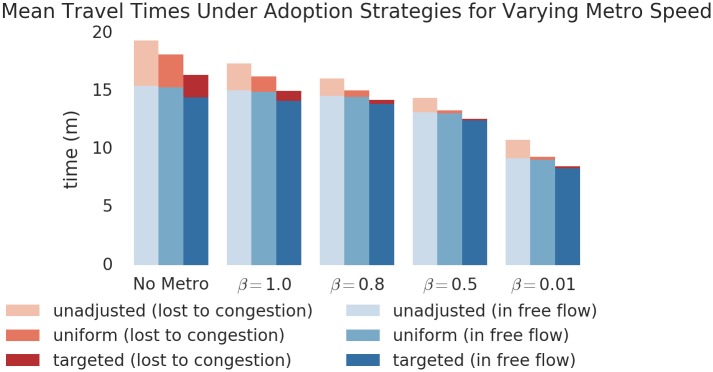
Performance evaluation of targeting strategies based on Δ_*od*_ for varying metro speeds *β*. The targeted approach achieves substantially shorter travel times, including large reductions in time lost to congestion. These benefits are persistent across metro speed, indicating that a targeted approach is beneficial in all cases.

The results in Fig 5 show that the targeted approach yields substantial time-savings over the uniform approach. In aggregate, travelers experience two kinds of reductions in travel time. First, adoption targeted to reduce congestion leads to substantial reductions in travel time lost to congestion. Secondly, reductions in congestion along key routes makes these routes more attractive to some travelers, who might otherwise have chosen a less-direct route to avoid traffic. Such travelers may therefore experience a reduction in free flow travel time as well.

These results indicate that, regardless of the speed of the metro, prioritizing areas with high Δ_*od*_ for connecting public transportation service could yield significant benefits for global congestion. Indeed, considering the estimated realistic case *β* = 0.8, the one minute difference in mean travel time between uniform and targeted strategies reflects 2.2 million person-minutes—or 4 person-years—saved every morning, and again every afternoon. The reduction in time spent specifically spent in congestion reflects 3.5 fewer person-years spent in congestion each day, a substantial boon for controlling environmental impact. We emphasize that these reductions are *in addition* to the reduced congestion experienced by the 15% of flow routed through public transit, who have been excluded from the analysis but would also benefit from improved traffic conditions.

## Discussion

It is striking that, even for an arbitrarily fast, uncapacitated metro, mean travel times decrease by just 43%. This is a relatively small drop considering that the metro in the limiting case effectively serves as a teleporter. This phenomenon is due to the spatially-organized, partially-optimal structure of travel demand. A shortest-path-seeking traveler will use the metro system if and only if the metro offers an improvement on driving directly there. In a model with uniform demand, commuters are modeled as choosing home and work locations without any preferences about how long they spend commuting. These models therefore result in a large number of very long trips. The longer a traveler’s commuting time, the more likely that the metro will offer a substantial improvement. In contrast, real commuters generally have a strong preference for a shorter commute. Thus, the average (network) distance between home and work in Riyadh under a uniform demand model is 22.3km, but is barely half that—11.6km—under observed demand. For a realistic commuter, it is therefore substantially less likely that the metro offers a substantial improvement in commuting time, leading to dampened effects. Modeling with observed travel demand thus changes both the quantitative and qualitative multiplex dynamics.

While increases in metro speed in our model lead to steady decreases in total travel time, the environmental benefits of extreme reduction are limited, as most of the benefit is achieved for metro speeds around *β* = 0.6, which is only slightly faster than our estimated realistic speed. This corresponds to an adoption level of *J*_*m*_ = 39%, which is just two thirds the adoption rate of a “teleporter” metro with very low *β*. This finding suggests that extreme levels of adoption may have fewer environmental benefits than might otherwise be expected. While more sophisticated modeling is necessary, it is intriguing that some subways may already be operating at speeds close to optimal for mitigating the impact of commuting traffic on emissions.

We have demonstrated a modeling and planning framework for a simplified urban transportation model consisting only of automobiles and the metro system. These methods are readily generalizable to more complex cases. For example, it would be possible to add layers for walking, buses, or other transportation modes. It is also possible to encode complex transfer logic by controlling the directionality of transfer edges between multiplex layers. While these systems pose more complex questions and therefore invite more complex analysis, the underlying methods and software can remain the same.
